# Effects of irisin on the dysfunction of blood–brain barrier in rats after focal cerebral ischemia/reperfusion

**DOI:** 10.1002/brb3.1425

**Published:** 2019-09-30

**Authors:** Peipei Guo, Zhao Jin, Huisheng Wu, Xinyi Li, Jianjuan Ke, Zongze Zhang, Qiu Zhao

**Affiliations:** ^1^ Department of Anesthesiology Zhongnan Hospital of Wuhan University Wuhan China; ^2^ Department of Gastroenterology Zhongnan Hospital of Wuhan University Wuhan China; ^3^ Hubei Clinical Center & Key Laboratory of Intestinal & Colorectal Diseases Wuhan China

**Keywords:** blood–brain barrier, cerebral ischemia/reperfusion, irisin, matrix metalloproteinase‐9

## Abstract

**Objective:**

To investigate whether irisin could protect against blood–brain barrier (BBB) dysfunction following focal cerebral ischemia/reperfusion in rats.

**Methods and Materials:**

Seventy‐two adult male Sprague Dawley rats weighing 280–320 g were randomly divided into three groups: sham operation group (S), focal cerebral ischemia/reperfusion group (FC), and irisin group (IR). Focal cerebral ischemia was induced by improved thread occlusion of right middle cerebral artery (MCAO) for 2 hr followed by reperfusion for 24 hr in rats. After 24 hr of reperfusion, the neurological evaluation was performed by the method of Longa's score. The histopathological changes were observed by HE staining. The brain water content was determined by detecting the wet weight and dry weight. The BBB permeability was assessed by fluorescence spectrophotometer and fluorescence microscopy for Evans blue (EB) extravasation. The activity and expression of matrix metalloproteinase‐9 (MMP‐9) in different groups were detected by immunohistochemical staining, Western blot, and gel gelatin zymography.

**Results:**

After MCAO, the neurological deficit scores, the infarct volume, the brain water content, and the EB content were higher in the FC group than those in the S group (*p* < .05). While after irisin treatment, these indicators mentioned above were lower than those in the IR group (*p* < .05). Moreover, the protein expression of MMP‐9 in the cortex increased significantly after MCAO, while irisin treatment could decrease the protein expression of MMP‐9 in the cortex (*p* < .05).

**Conclusion:**

Our data suggest that irisin can attenuate brain damage both morphologically and functionally and protect BBB from disruption after focal cerebral ischemia/reperfusion, which is highly associated with the inhibition of the expression and activity of MMP‐9 in the brain tissue.

## Graphical abstract

Irisin exerts neuroprotective effects against blood–brain barrier (BBB) breakdown, but the underlying mechanisms remain unknown. In this work, we evaluated the neuroprotective effects of irisin against BBB breakdown in a rat model of focal cerebral ischemia/reperfusion (MCAO). And all our results indicated that the potential underlying mechanism of neuroprotective effects of irisin might be associated with BBB permeability by regulating the expression and activity of matrix metalloproteinase‐9.

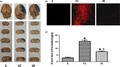

## 1 INTRODUCTION

Acute ischemic stroke (AIS) is one of the main reasons for morbidity and death worldwide, especially during perioperative period, and the incidence of AIS is generally 0.08%–0.4% in surgeries and anesthesia (Froehler et al., 2017; Wu, Guo, Jin, & Ke, 2018; Wu, Tang, Tai, & Yao, 2018). AIS is such an important cause for the deterioration of diseases and the rise of patients' mortality that it has grown to be a big concern of the medical community and society.

Acute ischemic stroke can lead to neurons damage. Worse still, restoring blood flow to the ischemic areas will partly aggravate ischemic brain damage and thus worsen the condition, and this pathological process is known as cerebral ischemia/reperfusion (Rosenberg, Estrada, & Dencoff, 1998). Cerebral ischemia/reperfusion is a complex and multifactorial process whose mechanism remains unclear at present. However, relevant studies have revealed that the destruction of blood–brain barrier (BBB) plays a vital role in the pathophysiology of cerebral ischemia/reperfusion (Fujimura et al., 1999; Kamada, Yu, Nito, & Chan, 2007; Reeves, Prins, Zhu, Povlishock, & Phillips, 2003; Strbian et al., 2008). Specifically, as the permeability of BBB increases, it promotes the formation of cerebral edema, which then expands brain volume, increases intracranial pressure, and fosters cerebral hernia and other adverse effects. Meanwhile, it aggravates inflammation reaction and apoptosis after cerebral ischemia by means of facilitating the inflammatory cells and inflammatory factors to pass BBB. This leads to deterioration of the disease and greatly influences clinical prognosis (Gardner & Ghorpade, 2003; Kamada et al., 2007; Strbian et al., 2008). Therefore, it is one of the most important measures to protect BBB and relieve cerebral edema for brain protection.

Matrix metalloproteinases (MMPs), the most important ECM‐degrading enzyme in vivo, plays an influential part in cerebral ischemia/reperfusion, especially when the expression and activity of matrix metalloproteinase‐9 (MMP‐9) increases in ischemic brain tissue. If so, MMP‐9 raises the permeability of BBB by degrading ECM so as to promote vasogenic cerebral edema, increase inflammatory cell infiltration, and promote inflammatory reaction and apoptosis, which aggravates cerebral ischemia/reperfusion (Fujimura et al., 1999; Kamada et al 2007; Reeves et al., 2003; Rosenberg et al., 1998). As a consequence, BBB dysfunction and ischemic brain damage can be alleviated partly by inhibiting the expression and activity of MMP‐9.

Irisin, a hormone accessible to the blood circulation, consists of a sequence of 112 amino acid residues. It is not only a compound muscle factor medium, but also a fat factor (Kim et al., 2018). An increasing proportion of evidence has shown that irisin regulates glucose and lipid metabolism in skeletal muscle and adipose tissue (Lee et al., 2015; Xin et al., 2016). In addition, previous studies have also confirmed that it also plays an important role in the pathophysiological development of pathological obesity, insulin resistance, type 2 diabetes, and metabolic diseases (Chen, Li, Liu, & Jia, 2016; Choi et al., 2013; Crujeiras et al., 2014; Moreno‐Navarrete, Ortega, Serrano, & Fernandez‐Real, 2013). Moreover, metabolic dysfunction is also associated with the pathogenesis of cardiovascular diseases. Our research team recently found that the irisin concentration levels in the serum of patients with ischemic stroke changed significantly from the early stage of stroke to postdischarge. We were equally shocked at the finding that the irisin content in the serum of patients with cerebral apoplexy was significantly statistically correlated with patients' prognosis (mainly from a cognitive perspective). Specifically, if patients with cerebral apoplexy had higher irisin concentration level in their serum, they would be expected with better prognosis and cognitive function. Contrarily, stroke patients with lower irisin concentration level in their serum had poor prognosis and cognitive function recovery (Wu, Guo, et al., 2018; Wu, Tang, et al., 2018). In this perspective, it is reasonable for us to speculate that treatment of irisin can exert certain impact upon the occurrence and development of cardiovascular and cerebrovascular diseases.

Based on the previous studies, this study is intended to explore the effects of irisin treatment on BBB permeability after focal cerebral ischemia/reperfusion in rats, and to explore its possible mechanism.

## 2 METHODS

### 2.1 Animals

Seventy‐two adult male Sprague Dawley rats, weighing 280–320 g, were obtained from Experimental Animal Center of Wuhan University. The rats were housed in a controlled environment (21 ± 2°C, 55 ± 5% relative humidity, 12‐hr light/dark cycle). Food and water were freely available to all rats throughout the experiment. Irisin was purchased from Cayman Chemical Company. All experimental procedures were performed under the National Institutes of Health Guidelines for the Care and Use of Laboratory Animals and approved by the Animal Care and Use Ethics Committee of Wuhan University.

### 2.2 Induction of focal cerebral ischemia/reperfusion

The improved thread occlusion of right middle cerebral artery (MCAO) model was carried out according to the previously described method with slight modification (Liu, Wang, Wang, & Zhu, 2014; Wu, Wang, Guo, & Shang, 2012; Zhai & Feng, 2018). In brief, rats were anesthetized with pentobarbital (50 mg/kg, i.p.). A midline cervical incision was made. Then, the right external carotid artery was located with its branches ligated. The induction of ischemia was performed by occluding right internal carotid artery with a thread. After 2 hr of transient MCAO, the nylon thread was removed to allow the reflow of blood through the right internal carotid artery (reperfusion). During this surgical procedure, the temperature was maintained at 37 ± 0.5°C by heated surgical platform. Rats in the S group received the same surgical procedure, except the right internal carotid artery was not occluded.

### 2.3 Experimental groups

Rats were randomly divided into three groups: (a) sham operation group (S), a healthy control group that subjected to sham operation; (b) focal cerebral ischemia/reperfusion group (FC), a FC group that underwent the occlusion of right middle cerebral artery (MCAO) for 2 hr followed by reperfusion for 24 hr; and (c) irisin group (IR), an IR group that underwent MCAO injury at 30 min after pretreatment with 10 μg/kg irisin intravenously.

### 2.4 Neurological deficit tests

Neurological evaluation (*n* = 24 in each group) was performed by the same examiner, who was blinded to the group assignment at 24 hr after reperfusion. The Longa test was used for this evaluation (Ye et al., 2010): grade 0, symptoms without neurological impairment (normal); grade 1, inextensibility of its left forepaw when lifting the rats' tail(mild); grade 2, circling to the left side while walking (moderate); grade 3, walking hard and leaning to the left (severe); and grade 4, cannot walk spontaneously (very severe).

### 2.5 Infarct volume measurement

The infarct volume measurement was carried out according to the previously described method after 24 hr of reperfusion (Wu et al., 2012; Ye et al., 2010). The brain tissue of rats (*n* = 6 in each group) was cut into coronal sections 2.0 mm thick. And 2% TTC (2,3,5‐triphenyltetrazolium chloride; Sigma) was used for staining these sections at 37°C for 20 min. Then, the sections were immersed in 4% paraformaldehyde overnight. The infarct area was divided and analyzed by the software system. The infarct tissue and noninfarct tissue were distinguished, and then, the total infarct volume was calculated by integrating the lesion area of all six measured sections. The size of infarct regions was expressed as the percentage of infarct volume to total brain volume.

### 2.6 Brain water content

The determination of the brain water content was evaluated by wet/dry method as described previously (Liu et al., 2014). At 24 hr after reperfusion, the rats (*n* = 6 in each group) were decapitated and its brains were removed quickly. The wet weight of ischemic hemispheres(right) was weighed using an electronic analytic balance, incubated in an oven at 110°C for 24 hr, and then reweighed immediately for the dry weight. The formula for calculating the brain water content is as follows:

$$\frac{\left(\text{wet}\;\text{weight}-\text{dry}\;\text{weight}\right)}{\text{wet}\;\text{weight}}\times 100\%$$

### 2.7 Evaluation of BBB permeability

The evaluation of BBB integrity (*n* = 6 in each group) was carried out by Evans blue (EB) content at 24 hr after reperfusion. EB dye (2% in saline, 4 ml/kg) was injected through the left femoral vein 1 hr before the brain was collected. Removal of intravascular dyes was performed by perfusion of precooled saline via left ventricle in rats. Then, bilateral cerebral hemispheres were separated quickly on ice and weighed separately and homogenized in 1.5 ml 50% trichloroacetic acid solution. After centrifugation (13,600 *g*, 20 min), the supernatant was diluted in anhydrous ethanol four times. The absorbance value was measured by fluorescence spectrophotometer (excitation wavelength 620 nm, emission wavelength 680 nm). The content of EB in brain tissue extract was quantified by microgram per gram of brain tissue. The leakage of EB dye was also observed with blue excitation light under a fluorescence microscope.

### 2.8 Expression and content of MMP‐9

#### 2.8.1 MMP zymography

The expression of MMP‐9 in the ischemic hemisphere was evaluated by the zymography method according to the manufacturer's instruction. Briefly, the prepared protein samples (40 μg) were loaded and separated on a 10% Tris‐glycine gel with 0.1% gelatin as a substrate. After electrophoresis, the gels were washed with distilled water and incubated for 24 hr at 37°C. After development, Coomassie brilliant blue staining solution was used to stain the gel. The gels were scanned by scanning densitometry, and the integral optical density (IOD) values were analyzed.

#### 2.8.2 Western blot

The prepared protein samples were extracted from the homogenate of the cortex of the ischemic hemisphere at 24 hr after reperfusion (*n* = 6 in each group). And then, the proteins were separated by Tris‐glycine SDS‐PAGE and transferred onto a nitrocellulose membrane. Membranes were blocked with 5% nonfat dry milk in TBS at 4°C overnight and then incubated with the primary antibodies against MMP‐9 (Abgent; 1:500). After incubation with the secondary antibodies at room temperature for 60 min, membranes were washed four times with TBS‐T. The protein expression for each sample was analyzed with the Quantity One image analysis software.

#### 2.8.3 Immunohistochemistry

Paraffin sections of brain tissue (*n* = 6 in each group) were taken and operated according to the instructions of immunohistochemical kit. Then, the results were analyzed. Five nonoverlapping visual fields around the infarct were randomly selected under 400‐fold light microscopy. Integral optical density (IOD) was measured by HMIAS‐2000 automatic medical color image analysis system (Champath Imaging Technology).

### 2.9 Statistical analysis

Data were expressed as mean ± *SD* using GraphPad 6 software (GraphPad Software). Data were analyzed by one‐way analysis of variance (ANOVA) followed by LSD multiple comparison tests as a post hoc comparison. A *p* value <.05 was considered statistically significant. Statistical Program for Social Sciences (SPSS) 19.0 was used in the study.

## 3 RESULTS

### 3.1 Neurological scores and Brain water content

**Figure 1:**
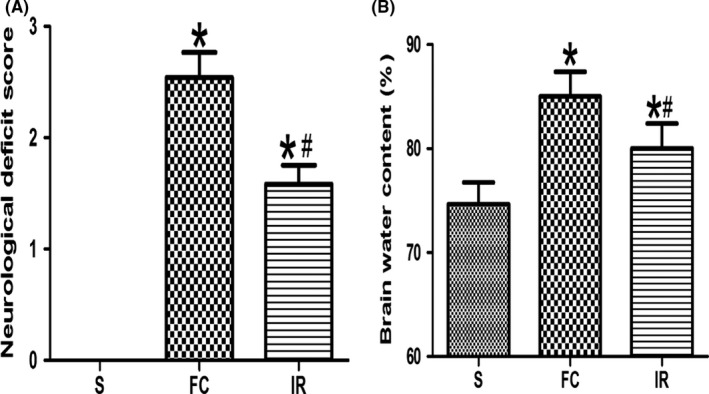
Effects of irisin on neurological deficit score and brain water content. (a) Comparison of neurological deficit score in different groups (*n* = 24). (b) Comparison of brain water content in different groups (*n* = 6). FC, focal cerebral ischemia/reperfusion group; IR, irisin group; S, sham operation group. Data are presented as the means ± *SD*. *p < .05 versus S group, #*p* < .05 versus FC group

After focal cerebral ischemia/reperfusion injury(MCAO), rats exhibited significant neurological deficit that is manifested as elevated neurological deficit score compared with that in the S group (2.54 ± 1.10 vs. 0.00 ± 0.00), while treatment of irisin significantly reduced neurological deficit score compared with the FC group (1.58 ± 0.83 vs. 2.54 ± 1.10; Figure 1a).

Compared with the S group, the brain water content significantly increased in the FC group (85.00 ± 5.83 vs. 74.67 ± 5.09). After treatment of irisin, the brain water content decreased in the IR group compared with that in the FC group (80.00 ± 5.93 vs. 85.00 ± 5.83; Figure 1b).

### 3.2 Infarction volume

**Figure 2:**
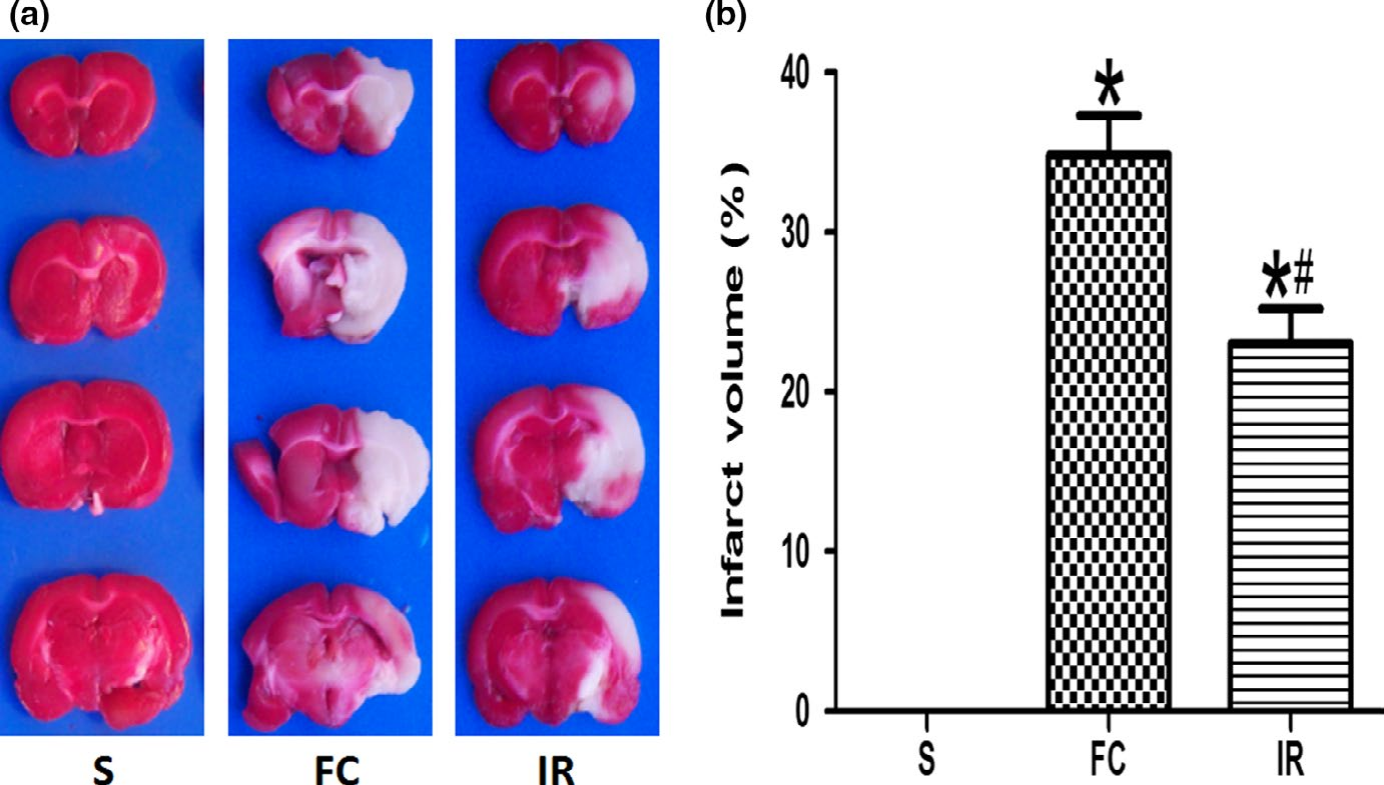
Effects of irisin on infarct volume. (a) Comparison of the slices with the largest infarct size infarction volume by TTC staining in different groups. (b) Comparison of the infarct volume in different groups. FC, focal cerebral ischemia/reperfusion group; IR, irisin group; S, sham operation group. Data are presented as the means ± *SD* (*n* = 6). *p < .05 versus S group, #*p* < .05 versus FC group

As shown in Figure 2, the infarcted regions in these sections were represented by the white‐colored areas. The cerebral infarcted regions were confined to the frontal cortex, parietal cortex, and temporal cortex in the right hemispheres, and the infarct size was calculated according to the ratio of the corrected infarct volume to the whole brain volume. The S group exhibited no damage, while in the FC group, an infarcted area was obviously observed. More than that, infarcted region of IR group narrowed down noticeably compared with the FC group (23.00 ± 5.40 vs. 34.83 ± 6.05; Figure 2a,b).

### 3.3 Evaluation of BBB permeability

**Figure 3:**
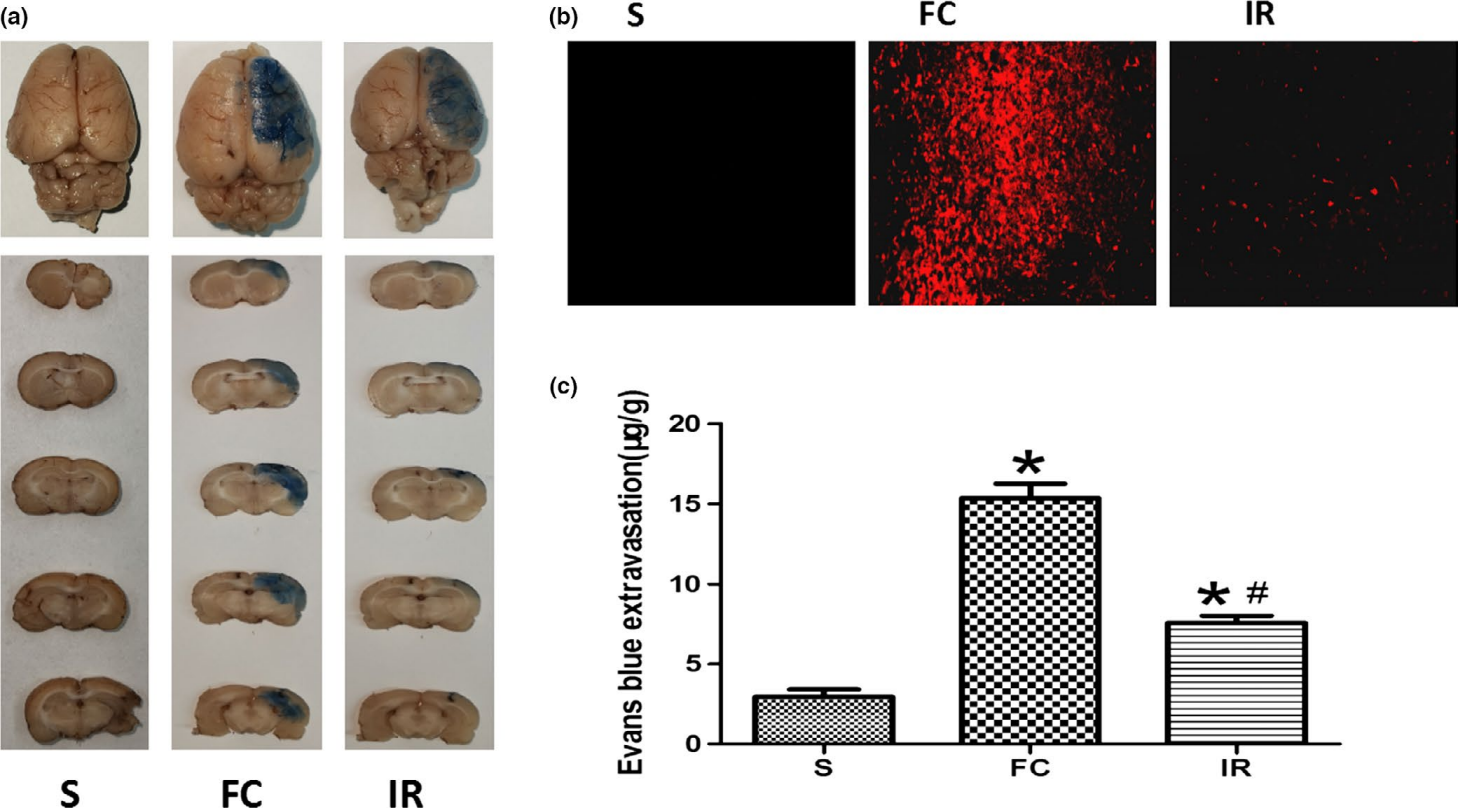
Effects of irisin on blood–brain barrier permeability. (a) Representative gross appearance of Evans blue (EB)‐stained brains from rats at 24 hr after reperfusion.(b) The leakage of EB in brain tissue observed by a fluorescence microscope. (c) Quantitative analysis of EB leakage in brain tissue. FC, focal cerebral ischemia/reperfusion group; IR, irisin group; S, sham operation group. Data are presented as the means ± *SD *(*n* = 6). *p < .05 versus S group, #*p* < .05 versus FC group

Evans blue was seen in the ischemic hemisphere of the coronal section of brain tissue (Figure 3a). EB can be seen as a red spot when irradiated by a blue laser under a fluorescence microscope (excitation wavelength 620 nm). It can reflect the leakage of EB in brain tissue according to the size and amount of the red spot. At 24 hr after reperfusion, a lot of red spots could be seen in the FC group and the EB content significantly increased compared with the S group (15.33 ± 2.25 vs. 2.95 ± 1.13), while after irisin treatment, the red spots were significantly reduced, and the EB content also followed this trend compared with the FC group (7.57 ± 1.11 vs. 15.33 ± 2.25; Figure 3b,c).

### 3.4 Expression and content of MMP‐9

**Figure 4:**
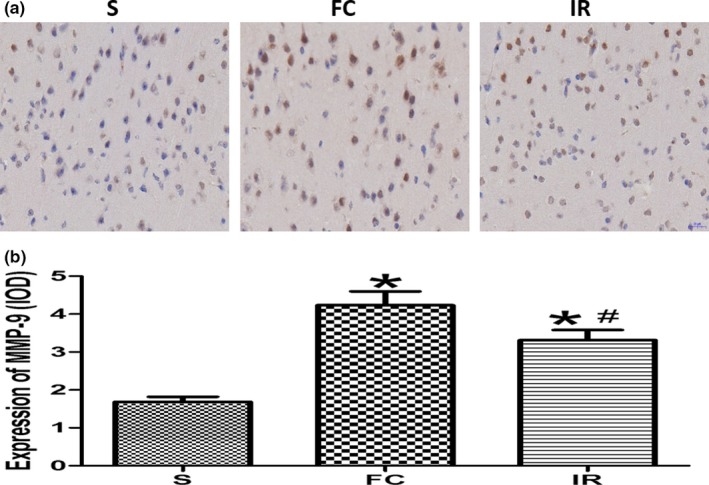
Effects of irisin on the expression of matrix metalloproteinase‐9 (MMP‐9) by immunohistochemical staining. (a) Comparison of the slices with the expression of MMP‐9 by immunohistochemical staining in different groups. (b) Quantitative analysis of the MMP‐9 expression in different groups. FC, focal cerebral ischemia/reperfusion group; IR, irisin group; S, sham operation group. Data are presented as the means ± *SD *(*n* = 6). *p < .05 versus S group, #*p* < .05 versus FC group

Immunohistochemical staining of brain section showed that MMP‐9 was mainly expressed in the cytoplasm and the positive cells were brown (Figure 4a). The amount of positive cells in the peripheral ischemic area in the FC group and IR group increased compared with that in the S group (Figure 4a). This increased expression of MMP‐9 was also confirmed by IOD analysis (4.23 ± 0.89 vs. 1.68 ± 0.33, 3.32 ± 0.64 vs. 1.68 ± 0.33; Figure 4b), while in the IR group, the number of positive cells decreased compared with that in the FC group (Figure 4a), which was also consistent with the decreased expression of MMP‐9 done by IOD analysis (3.32 ± 0.64 vs. 4.23 ± 0.89; Figure 4b).

**Figure 5:**
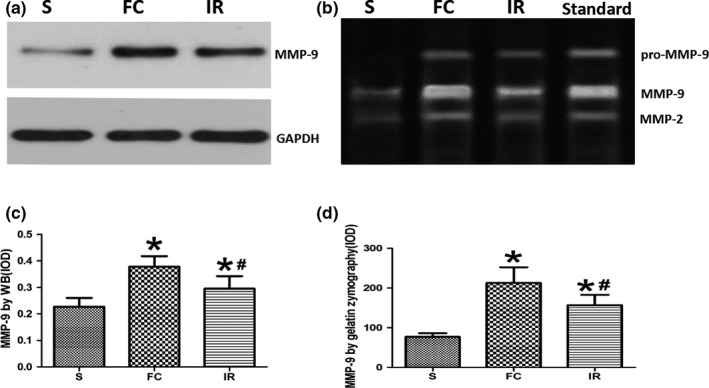
Effects of irisin on expression of matrix metalloproteinase‐9 (MMP‐9) by different analytical method. (a, c) Comparison of the expression of MMP‐9 by Western blot in different groups. (b, d) Comparison of the expression of MMP‐9 by gelatin zymography in different groups. FC, focal cerebral ischemia/reperfusion group; IR, irisin group; S, sham operation group. Data are presented as the means ± *SD *(*n* = 6). *p < .05 versus S group, #*p* < .05 versus FC group

At 24 hr after reperfusion, Western blot analysis showed that the expression of MMP‐9 increased in the FC group and IR group compared with the S group. Meanwhile, the treatment of irisin significantly downregulated the expression of MMP‐9 compared with the FC group (*p* < .05; Figure 5a,c).

At 24 hr after reperfusion, our zymography analysis revealed that the expression of activated MMP‐9 increased in the FC group and IR group, while the treatment of irisin significantly lightened the intensity of the MMP‐9 band. There was no significant change in MMP‐2 expression activity in three groups (*p* < .05; Figure 5b,d).

## 4 DISCUSSION

Irisin, a hormone released from skeletal muscle, has recently been illustrated to be a polypeptide 24 of 112 amino acids secreted by skeletal muscle cells during skeletal muscle movement. As skeletal muscle moves, the transcription factor PPAR7 in skeletal muscle cells assists activating factor 1α (PGC‐1α) and enhances its activity. This enhances gene expression activity of FNDC5 (fibronectin type III domain protein), and irisin is just the extracellular segment of FNDC5. When irisin exfoliates and is released into blood, it then exerts various effects. It was found that the irisin can reduce the blood sugar level of diabetic mice and improve the vasodilatation ability of mice (Lee et al., 2015; Moreno‐Navarrete et al., 2013; Xin et al., 2016). Clinical studies indicated that the serum levels of irisin in patients with type 2 diabetes were 30% lower than the normal level, so the irisin becomes a highlight and anticipation in the treatment of type 2 diabetes (Choi et al., 2013; Crujeiras et al., 2014). Due to the correlation among cerebrovascular diseases, pathological obesity, and diabetes, we intend to observe how the exogenous treatment of irisin will influence the permeability of BBB after focal cerebral ischemia/reperfusion in rats in the present study.

The method of middle cerebral artery occlusion (MCAO) was used in this study to establish a middle cerebral artery ischemia and reperfusion model in rats. It is a classical and commonly used animal model for ischemic cerebrovascular disease. It is advantageous in many aspects; that is, it is easy to operate, brings small wounds to animals, has high repeatability, and produces cerebral ischemic lesions with severe BBB destruction and significant brain edema (Liu et al., 2014). Therefore, the MCAO model is suitable for this study.

Currently, the key treatment of AIS and other ischemic stroke is to restore the blood flow to the ischemic brain tissue in time, but reperfusion can also lead to reperfusion injury, leading to cerebral hemorrhage and brain edema (Yang & Betz, 1994). The destruction of BBB brought by ischemia/reperfusion is one of the essential reasons for reperfusion injury. The mechanism remains unclear. By studying the expression of gelatinase in the brain from the sixth hour to 30th day after cerebral ischemia, Romanic, White, Arleth, Ohlstein, and Barone (1998) found that the expression of MMP‐9 increased significantly at 12 hr after ischemia, peaked at 24 hr, and lasted for several days. Such expression majorly occurred in the ischemic areas and in the neutrophils and endothelial cells around the ischemic peripheral regions. Five days later, it appeared in macrophages in the infarcted area and decreased to zero after 15 days. The administration of neutralizing MMP‐9 monoclonal antibody significantly reduced cerebral infarction. This suggests that the increase in early MMP‐9 has a close correlation with cerebral ischemic injury. Compared with MMP‐9, the expression of MMP‐2 lagged behind and peaked on the fifth day after ischemia. This is basically consistent with the cycle of brain edema after ischemic stroke in patients. Related clinical studies have found that the expression of MMP‐9 in the infarcted area and its surrounding area significantly increased after cerebral infarction in patients. It was also found that MMP‐9 was mainly expressed in perivascular areas, infiltrating neutrophils and activated microglia in the infarct center. In comparison, in the peripheral region of infarction, MMP‐9 was mainly expressed in macrophages. This suggests that MMP‐9 has a close correlation with ischemic brain injury and secondary cerebral edema (Rosell et al., 2006). At the second week since onset, MMP‐9 expression decreased to the basic level. This indicates that MMP‐9 is associated with clinical course and that MMP‐9 is expected to be a target for clinical treatment and help determine the prognosis of patients with ischemic cerebrovascular disease (Romanic et al., 1998; Rosell, Cuadrado, Ortega‐Aznar, & Joan, 2008). It is found that active MMP‐9 existed in the brain of mice appeared at 3 hr after reperfusion, which is consistent with the opening of BBB (Fujimura et al., 1999). Further studies found that the opening of BBB after cerebral ischemia was bidirectional. The first‐time opening of BBB may be related to the expression level of MMP‐2, and the second one was mainly related to the rise of MMP‐9 level. BB‐1101, the inhibitor of MMP, inhibits the first opening of BBB and the development of cerebral edema, suggesting the high correlation between MMPs and the opening of BBB together with the formation of cerebral edema (Rosenberg et al., 1998). Evidences found that the expression and activity of MMP‐9 in the ischemic brain tissue increased significantly in the early stage of cerebral ischemia/reperfusion, while MMP‐2 showed no significant change (Liu, Hendren, Qin, & Liu, 2009). It is thus speculated that the degradation of extracellular matrix caused by MMP‐9 is an important mechanism of BBB destruction in cerebral ischemic injury.

The destruction of BBB is an important pathological basis of cerebral ischemia/reperfusion injury (Fujimura et al., 1999; Kamada, Durukan, Pitkonen, & Tatlisumak, 2008; Kamada et al., 2007). The destruction of BBB leads to its increased permeability so as to promote the formation of vasogenic cerebral edema and is conducive to inflammatory cells and harmful substances into the brain tissue. Therefore, it is an important factor leading to the deterioration of the disease, affecting clinical prognosis (Kamada et al., 2008; Rosell et al., 2008). This can explain why reducing BBB permeability and improving brain edema is one of the key measures for clinical treatment. EB is a small molecule indicator. Normally, it fails to pass BBB after it binds to plasma proteins. Only in the pathological state where the BBB is destructed and its permeability increases, EB is able to enter the brain parenchyma through the damaged BBB. Most noteworthy, the EB content in the brain tissue is positively correlated with the degree of destruction of BBB. So, the degree of BBB damage can be ensured by measuring the EB content in brain tissue (Kaya, Gulturk, Elmas, & Sivas, 2004). In this study, the results showed that the EB content in brain tissue of rats in the FC group increased compared with that in the S group at 24 hr after reperfusion. The finding indicates that at that moment, the plasma protein leaked out in the brain tissue and that BBB was obviously damaged, resulting in the significant increase in its permeability. This is consistent with the results of the BBB injury after the cerebral ischemia/reperfusion of rats in previous studies (Guo, Cox, Mahale, & Ding, 2008).

Results of this study showed that rats appeared to be in obvious neurological deficit at 24 hr after reperfusion. The results of TTC showed clear‐bordered pale infarct focus in the right frontal, parietal, and caudate nucleus of the brain in the FC group. The quantitative results of cerebral infarction volume increased and that the water content and the EB content in the brain tissue increased significantly in the FC group. These findings suggest that the rat model of focal cerebral ischemia/reperfusion is successful in this study. They also indicate that BBB was severely damaged, and thus, its permeability significantly increased, causing brain edema at 24 hr after reperfusion. Meanwhile, the neurological deficit score decreased, the volume of infarction decreased, the interstitial edema significantly alleviated, and the water content and EB content in brain tissue decreased significantly in the IR group. These findings suggest that irisin can improve the neurological function and reduce the BBB injury.

Matrix metalloproteinase‐9, also known as gelatinase B, can be expressed in a variety of cells, such as neurons, endothelial cells, activated astrocytes, microglia, and infiltrating neutrophils (Candelario‐Jalil, Yang, & Rosenberg, 2009). During cerebral ischemia/reperfusion, increased expression and activity of MMP‐9 is one of the vital mechanisms that destruct BBB, promote the formation of brain edema, and aggravate ischemic brain damage (Fujimura et al., 1999; Kamada et al., 2007). Asahi et al. (2001) found that MMP‐9 knockout mice showed significantly lower permeability of BBB, lower white matter damage, and smaller infarct volume in cerebral ischemia/reperfusion than wild mice. The mechanism may be correlated with inhibiting the degradation of tight junction proteins and myelin basic proteins between endothelial cells. Hu et al. (2009) employed gene silencing technology to inhibit the expression and activity of MMP‐9 in the brain. They found that it could significantly reduce the infarct volume, brain water content, mortality, and accompanied neurological deficit after cerebral ischemia in rats. All these studies directly confirmed that the destruction of BBB is closely related to MMP‐9. That is, the inhibition of MMP‐9 expression and activity protects BBB, reduces vasogenic cerebral edema and infarct volume, and promotes neurological recovery. The immunohistochemical results of this study showed that the amount of MMP‐9 positive cells was very low in rat brain tissue of the S group. Meanwhile, the amount of MMP‐9‐positive cells was abundantly existed in neurons, endothelial cells, gliacytes, and infiltrating neutrophile granulocytes in the FC group, which is consistent with the results of Rosenberg, Cunningham, Wallace, and Gearing (2001), while treatment of irisin significantly reduces the number of MMP‐9‐positive cells in the IR group. The results showed that the activity of MMP‐9 and MMP‐2 was quite low in the S group by the method of gelatin zymography. The activity of MMP‐9 was significantly enhanced after MCAO, but the activity of MMP‐2 had no significant change. Treatment of irisin noticeably inhibited the activity of MMP‐9. The results of Western blot showed that the expression of MMP‐9 in the brain tissue of the S group was very low, while the expression of MMP‐9 in the FC group was higher than that in the S group at 24 hr after reperfusion. After treatment with irisin, the expression of MMP‐9 significantly decreased, which was consistent with the changes in the BBB permeability and the brain water content. All these results suggest that irisin may alleviate the damage of BBB and cerebral edema by inhibiting the expression and activity of MMP‐9.

In conclusion, the present study indicates that irisin can protect BBB against disruption and alleviate brain edema after focal ischemia/reperfusion injury. The mechanism underlying these neuroprotective effects may be associated with affecting BBB permeability by regulating the expression and activity of MMP‐9. Based on the results of previous studies, these findings may provide a theoretical basis for clinical diagnosis and treatment of AIS.

## CONFLICT OF INTEREST

None declared.

## Data Availability

The data that support the findings of this study are available from the corresponding author upon reasonable request.
